# Supplementary material for: α-catenin phosphorylation is actomyosin-sensitive and required for epithelial barrier functions through Afadin
Source: bioRxiv. 2025 Aug 22:2025.08.21.671625. Preprint. [Version 1] doi: 10.1101/2025.08.21.671625 (PMC12393524; doi:10.1101/2025.08.21.671625)
Supplement: 1 [file NIHPP2025.08.21.671625V1-supplement-1.pdf]

bioRxiv preprint doi: https://doi.org/10.1101/2025.08.21.671625; this version posted August 22, 2025. The copyright holder for this preprint (which was not certified by peer review) is the author/funder, who has granted bioRxiv a license to display the preprint in perpetuity. It is made available under aCC-BY 4.0 International license.

**Figure S1: Restoration of GFP-α-cat mutants in α-cat CRISPR-KO MDCK cells.** (**A**) Immunoblot of MDCK cells restored with GFP-tagged forms of α-cat via lentiviral delivery. Total-α-cat antibody detects similar α-cat expression across restored cell lines. Specific phospho-sites removed in 4A (phospho-mutant) and 4E (phospho-mimic) are not detected by the phospho-specific antibody. Ratio of phospho-to-total α-cat was quantified and standardized to WT. β-tubulin was used as loading control. α-cat restored lines were sort-matched by flow cytometry and periodically blotted to ensure against expression drift. (**B**) Schematic of GFP-α-cat mutant forms used in this study.

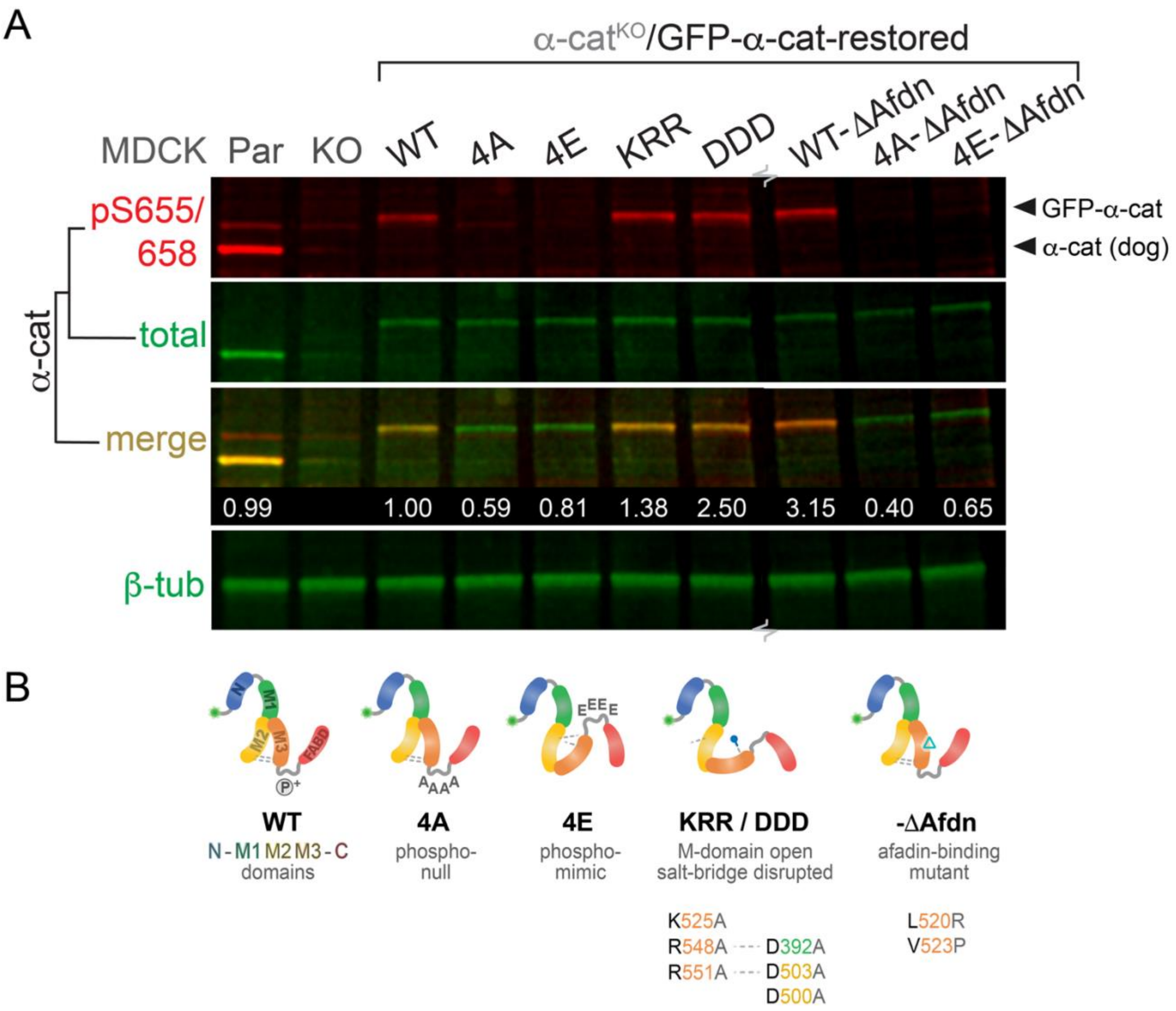

bioRxiv preprint doi: https://doi.org/10.1101/2025.08.21.671625; this version posted August 22, 2025. The copyright holder for this preprint (which was not certified by peer review) is the author/funder, who has granted bioRxiv a license to display the preprint in perpetuity. It is made available under aCC-BY 4.0 International license.

**Figure S2: α-cat P-linker phospho-mimic promotes M-domain accessibility via in solution kinase assay.** (**A**) Schematic of α-cat constructs with phospho-proteomic defined P-linker S/T residues replaced by non-modifiable phospho-mimic (aspartate, (D) or glutamate (E)) and phospho-mutant (alanine, (A)) residues. We previously showed that CK2 phosphorylates α-cat at S641, whereas CK1 sequentially phosphorylates α-cat at S652, S655 and T658 (Escobar et al., 2015). For loss-of-phosphorylation studies, T654 was also mutated, because pT654 could not be confidently distinguished from pS655 by mass spectrometry (Harvard Taplin Facility). Since CK1 typically phosphorylates proteins at S/TxxS/TxxS/T residue spacing, we favor the phospho-scheme shown in A (red arrows), but it is formally possible T654 is modified non-canonically. Thus, these early in vitro phosphorylation studies relied on P-linker 5-residue mutant (α-cat-5A, green) and mimic proteins (α-cat-5D, magenta), respectively.  An α-cat 9-residue total phospho-mutant (α-cat 9E or P-null, blue), prevented modification of 4 additional phospho-sites localizing to α-cat's M-domain (S453, S455, S507 and T634). These latter sites were identified in our original phospho-proteomic mass spectrometry analysis of α-cat[66] (Harvard Taplin Facility). (**B-E**) Kinase activity is measured by the incorporation of $\gamma^{32}$P-incorporation over time. After incubation, GST-purified α-cat was subjected to gel electrophoresis, Coomassie staining, gel drying and exposure to film. (**D-E**) Kinase accessibility was quantified (**B-C**) by standardizing the full-length (FL) $\gamma^{32}$P-containing α-cat bands (film exposure, grayscale image) to total α-cat detected by Coomassie stain (lower image, purple). Normalized values from 3 experiments were plotted and compared by multiple t-tests; *P<0.05, **P<0.01. (**F-G**) α-cat M-domain residues mutagenized in construct P-null 9E (blue). (**F**) Full length α-cat crystal structure highlighting locations of S/T residues in M2 and M3. All mutagenized S/T residues were evaluated for potential confounding salt-bridge interactions with nearby residues.  (**G**) Zoomed-in view of the residues in M2, with nearby sidechains showing no charge-based interactions. Evidence these residues (S453, S455, S507 and T634) are more accessible to in vitro phosphorylation in the α-cat 5E versus α-cat 5A mutant suggests that α-cat P-linker modification allosterically alters the M-domain.

bioRxiv preprint doi: https://doi.org/10.1101/2025.08.21.671625; this version posted August 22, 2025. The copyright holder for this preprint (which was not certified by peer review) is the author/funder, who has granted bioRxiv a license to display the preprint in perpetuity. It is made available under aCC-BY 4.0 International license.

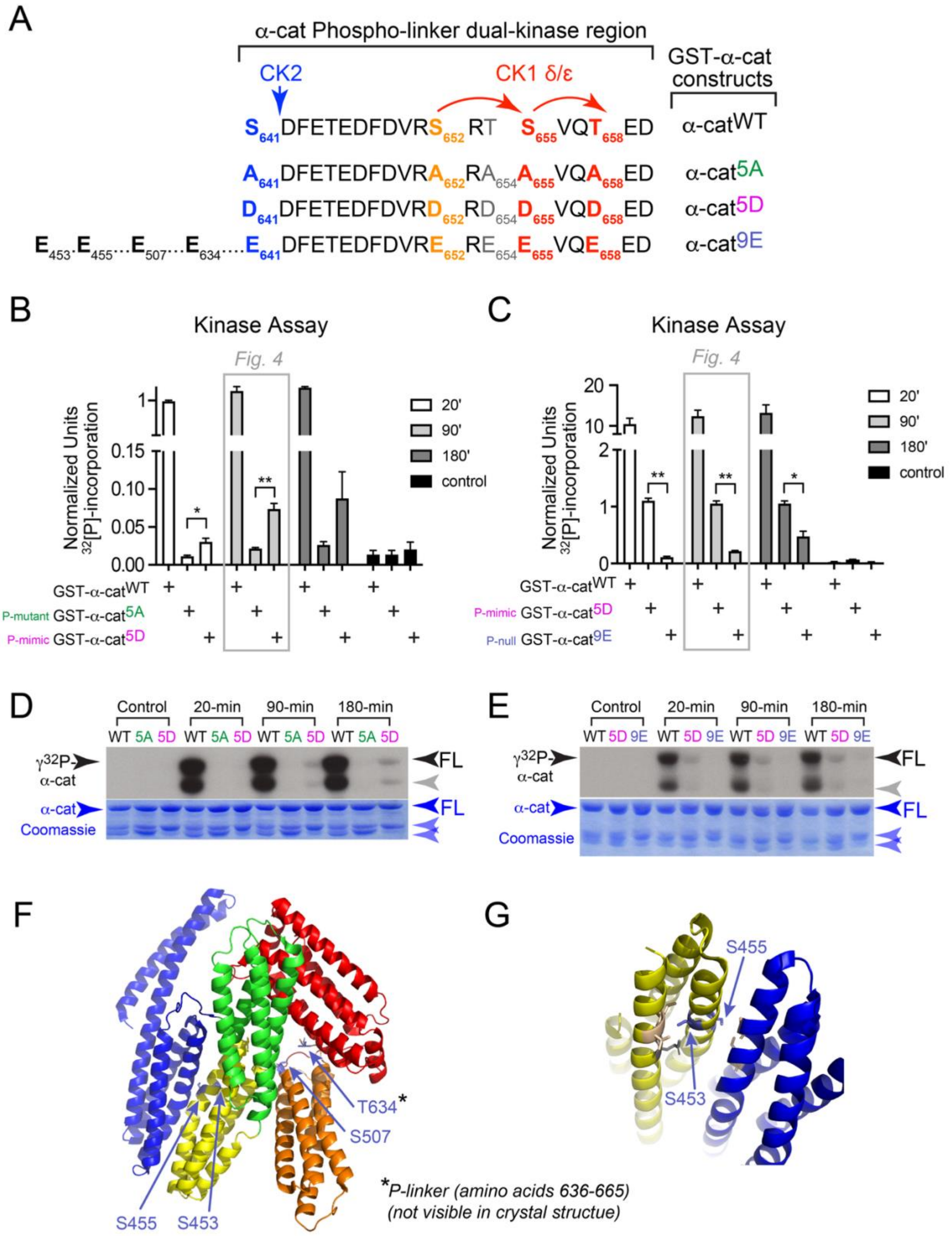

bioRxiv preprint doi: https://doi.org/10.1101/2025.08.21.671625; this version posted August 22, 2025. The copyright holder for this preprint (which was not certified by peer review) is the author/funder, who has granted bioRxiv a license to display the preprint in perpetuity. It is made available under aCC-BY 4.0 International license.

**Figure S3: Modeling of the α-cat M3/Afadin-binding interaction. A**) NMR titration analysis of $^{15}$N-labeled αN-cat-M3 interaction with unlabeled Afadin-CC-ABR. Overlay of $^{1}$H-$^{15}$N HSQC spectra of $^{15}$N-αN-cat-M3 with unlabeled Afadin-CC-ABR at various molar ratios are shown, indicating Afadin binding by select αN-cat-M3 residues. These data were used to validate the binding interface in Fig. 5C. **B**) Multiple sequence alignment of helix 1 in αE-cat (*CTNNA1*) M3-domain demonstrates preserved residues across species and isoforms. **(C-D)** Predictions from molecular docking simulations between Afadin and α-cat WT or ΔAfdn. (**C**) α-cat M3 helix 1 is predicted to interact with Afadin through residues L520 and V523. (**D**) Afadin and α-cat ΔAfdn docking predictions yield misaligned interactions.

bioRxiv preprint doi: https://doi.org/10.1101/2025.08.21.671625; this version posted August 22, 2025. The copyright holder for this preprint (which was not certified by peer review) is the author/funder, who has granted bioRxiv a license to display the preprint in perpetuity. It is made available under aCC-BY 4.0 International license.

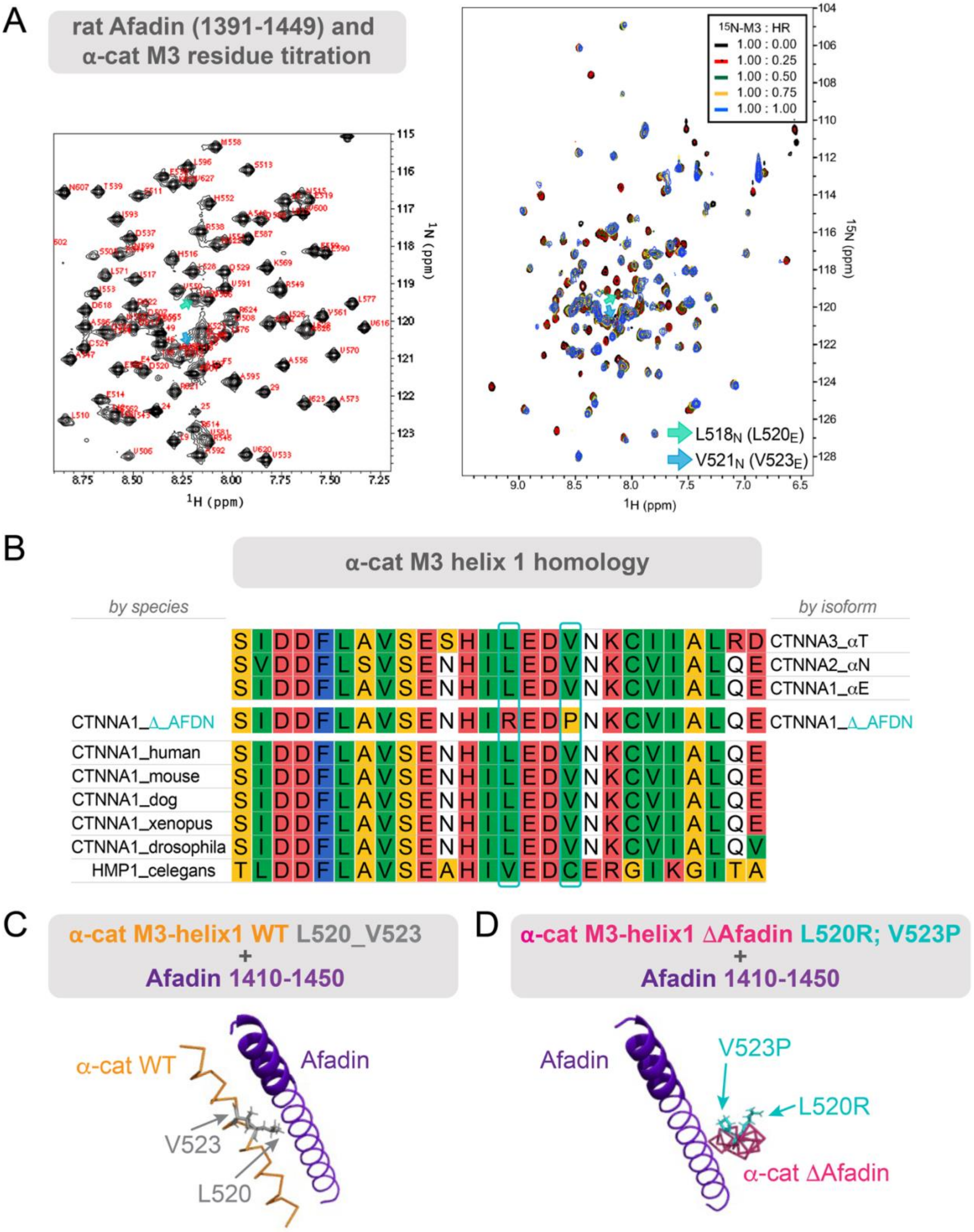

bioRxiv preprint doi: https://doi.org/10.1101/2025.08.21.671625; this version posted August 22, 2025. The copyright holder for this preprint (which was not certified by peer review) is the author/funder, who has granted bioRxiv a license to display the preprint in perpetuity. It is made available under aCC-BY 4.0 International license.

**Fig. S4: Afadin does not substantially co-immunoprecipitate with α-cat in MDCK epithelial cell lysates.** Immunoblot validation of α-cat WT and ΔAfadin (Afdn) protein expression in α-cat CRISPR KO MDCK cells. Parental MDCK cells shown (left-most lane). Note that despite robust GFP-α-cat affinity precipitation in both cell lines, Afadin co-immunoprecipitation was weak.

A

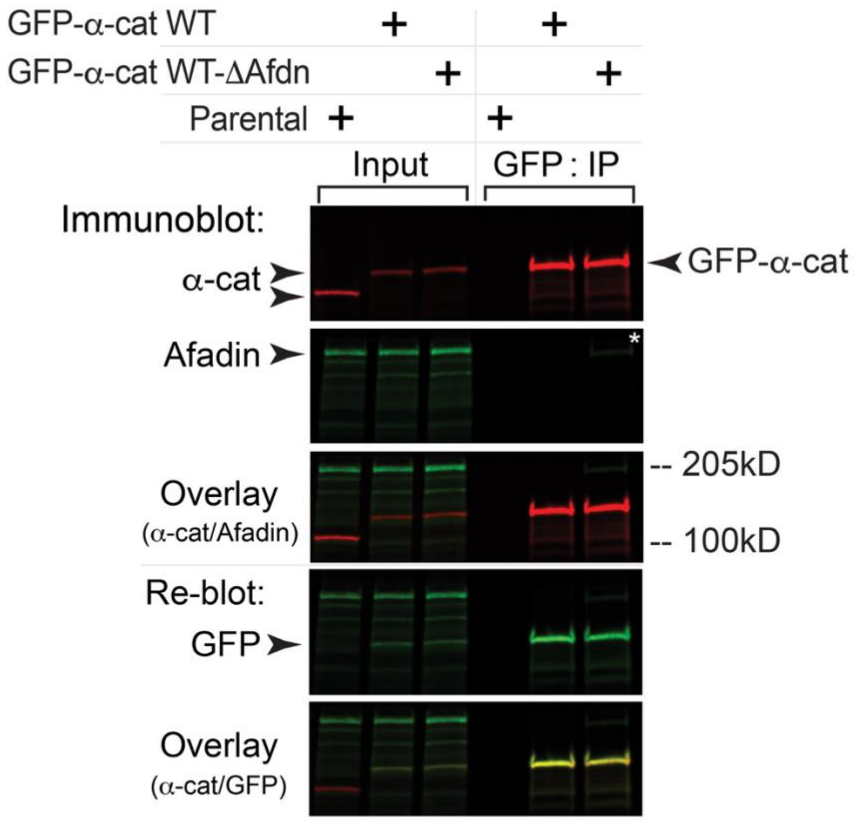

bioRxiv preprint doi: https://doi.org/10.1101/2025.08.21.671625; this version posted August 22, 2025. The copyright holder for this preprint (which was not certified by peer review) is the author/funder, who has granted bioRxiv a license to display the preprint in perpetuity. It is made available under aCC-BY 4.0 International license.

**Figure S5: α-cat M3-Δ-Afadin binding mutant does not phenocopy Afadin KO MDCK cells matured on glass.**

(**A**) Confocal images of glass coverslip plated MDCK monolayers with restored GFP-α-cat WT and Δ-Afdn forms fixed and immuno-stained for Afadin (magenta) and F-actin (phalloidin, cyan). Native GFP-α-cat is shown in green. *En face* images are maximum intensity projections (M.I.P.). Scale bar = 20μm. Dotted boxes correspond to expanded inset views (right). Arrows point to multi-vertex junctions that appear to show less α-cat/Afadin colocalization for the α-cat Δ-Afdn mutant. This is especially clear for the apical most portion of bi-cellular junctions (single apical section), where white arrows show better colocalization between α-cat WT and Afadin versus the α-cat Δ-Afdn mutant, where separation of α-cat (green arrows) and Afadin signal (magenta arrows) is shown. Asterisk (yellow) shows corresponding cell in lower mag view; note that views are rotated. (**B**) Confocal images (maximum intensity projection) of Afadin KO MDCK cells grown on glass. Dotted box corresponds to expanded insets (right). Note that F-actin recruitment to cell-cell junctions is reduced in Afadin KO cells (asterisks), as previously shown[15]. Note F-actin recruitment is not obviously altered between α-cat WT and α-cat Δ-Afdn mutant cells (A, above insets). (**C**) Quantification of F-actin junction intensity from images in A and B, using 1μm circular ROIs taken from bicellular junctions, subtracting adjacent cytoplasm signal. Normalized fluorescence was plotted, with symbols corresponding to 1 biological experiment analyzing 50-75 junctions/construct. ****P<0.0001 by ANOVA with Tukey's multiple comparison. (**D**) Quantification of α-cat/Afadin colocalization differences seen in A across multiple junctions. Intensity correlation was analyzed by Pearson's (WT r = 0.6430, ****p < 0.0001; α-cat Δ-Afdn r = 0.4298, ****p < 0.0001). This suggests weaker colocalization between α-cat Δ-Afdn mutant and Afadin at zAJs than α-cat WT and Afadin.

bioRxiv preprint doi: https://doi.org/10.1101/2025.08.21.671625; this version posted August 22, 2025. The copyright holder for this preprint (which was not certified by peer review) is the author/funder, who has granted bioRxiv a license to display the preprint in perpetuity. It is made available under aCC-BY 4.0 International license.

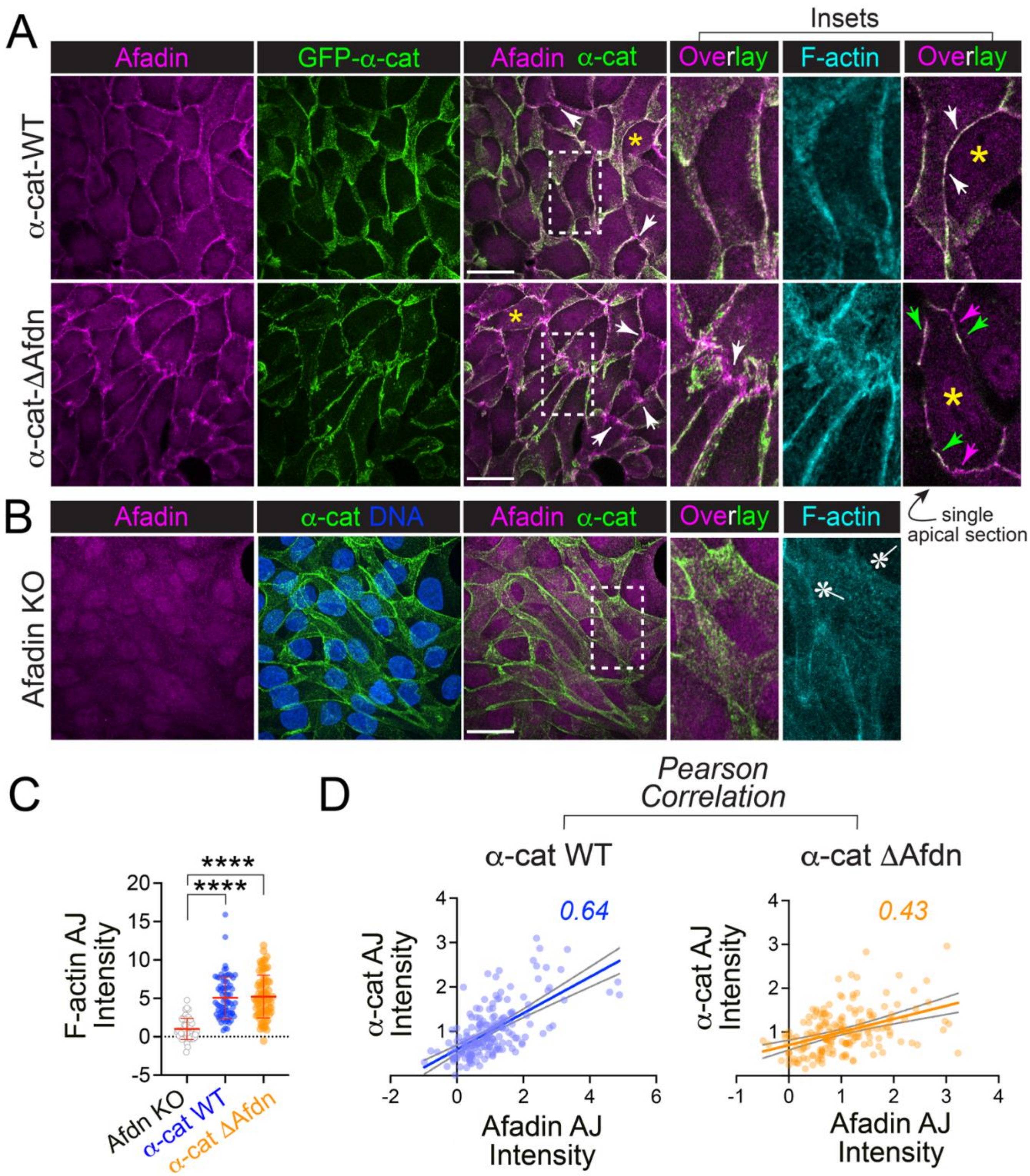


744

bioRxiv preprint doi: https://doi.org/10.1101/2025.08.21.671625; this version posted August 22, 2025. The copyright holder for this preprint (which was not certified by peer review) is the author/funder, who has granted bioRxiv a license to display the preprint in perpetuity. It is made available under aCC-BY 4.0 International license.

**Figure S6: α-cat M3-Δ-Afadin binding mutant does not phenocopy Afadin KO MDCK cells matured on filters.**

(**A**) Confocal images of MDCK monolayers matured on filters with restored GFP-α-cat WT and Δ-Afdn forms fixed and immuno-stained for Afadin (magenta) and F-actin (phalloidin, yellow). Native GFP-α-cat is shown in green. *En face* images are single x-y optical slices. White arrows show multi-vertex junctions, which clear gaps for the α-cat Δ-Afdn mutant. Scale bar = 5μm. Orthogonal (x-z) views are shown to right; section marked by white arrowheads in overlay image. Asterisk shows recessed apical junctions extending basally in the α-cat Δ-Afdn mutant. (**B**) Confocal images (maximum intensity projection) of Afadin KO MDCK cells matured on filters (2-weeks), fixed and immuno-stained for Afadin (magenta). Arrows show normal (un-recessed) multi-vertex junctions. Scale bar = 20μm. Note that on filters, we did not detect an obvious loss of F-actin recruitment to zAJ (F-actin, blue; α-cat, yellow), although Afadin KO cells showed greater capacity to form intra-epithelial (ectopic) lumens (lower left, schematic).

bioRxiv preprint doi: https://doi.org/10.1101/2025.08.21.671625; this version posted August 22, 2025. The copyright holder for this preprint (which was not certified by peer review) is the author/funder, who has granted bioRxiv a license to display the preprint in perpetuity. It is made available under aCC-BY 4.0 International license.

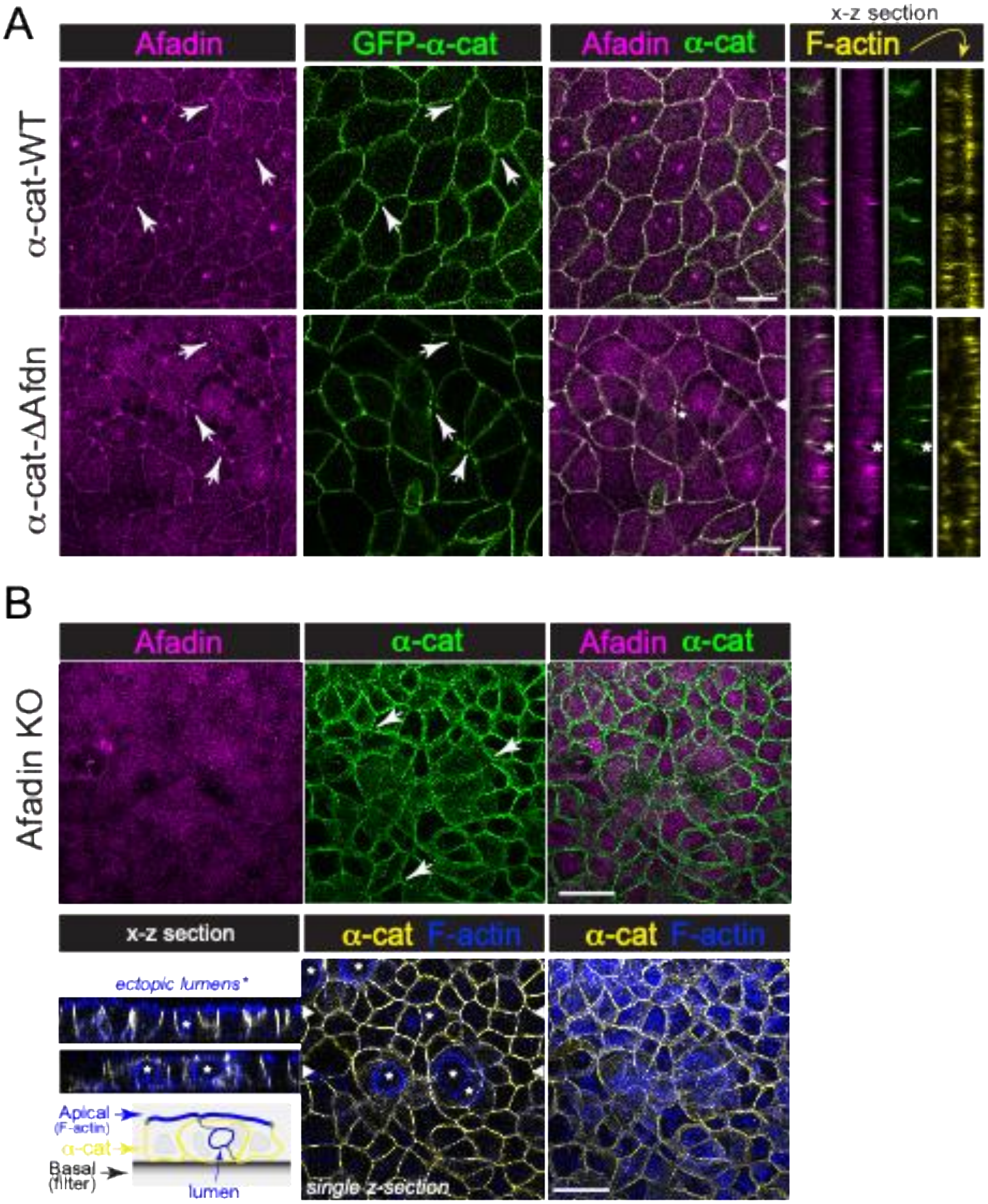


759

bioRxiv preprint doi: https://doi.org/10.1101/2025.08.21.671625; this version posted August 22, 2025. The copyright holder for this preprint (which was not certified by peer review) is the author/funder, who has granted bioRxiv a license to display the preprint in perpetuity. It is made available under aCC-BY 4.0 International license.

**Figure S7: Immunoblot validation of *Ctnna1*-phospho-mutant knock-in mouse line by tissue.** (**A**) Lysates prepared from *Ctnna1*$^{WT/WT}$ (Wild-type, WT), *Ctnna1*$^{WT/4A}$ (heterozygous, HET) or *Ctnna1*$^{4A/4A}$ (homozygous, HOMO) mouse kidney show that α-cat phosphorylated at S641 or S655/T658 are reduced or ablated in kidney lysates prepared from HET or HOMO mutant mice relative to a littermate WT control. Immunoblot detection using a LiCOR imaging system where nitrocellulose was simultaneously incubated with antibodies to phospho-α-cat (green) or total α-cat (red) (**B**) Lysates prepared cerebellum corresponding to cerebrums analyzed in Figure 7 and subjected to immunoblotting as in A. While detection of α-cat phosphorylated at S641 is completely blocked in this tissue, pS652 is reduced and pS655/pT658 immunodetection persists in the *Ctnna1*$^{4A/4A}$ (HOMO) condition. Since brain (cerebellum and cerebrum) expresses the neural isoform of α-cat, *Ctnna2*/αN-catenin, we affirmed expression of this isoform in C. (**C**) Immunoblot with antibody to αN-cat, showing abundant expression of this protein. (**D**) Graph quantification of α-cat pS641, pS652 and pS655/pT658 detection from brain lysates (cerebellum and cerebrum) of two different animals. Collectively, these data show that the *Ctnna1*$^{4A/4A}$ HOMO-knock-in mouse completely blocks phosphorylation of αE-cat pS641, pS652 and pS655/pT658 in tissues that only express αE-cat/Ctnna1 (A, kidney). Tissues that express other α-cat isoforms, such as brain, reveal detection of identical phospho-sites in these isoforms (αN-cat; αT-cat).

bioRxiv preprint doi: https://doi.org/10.1101/2025.08.21.671625; this version posted August 22, 2025. The copyright holder for this preprint (which was not certified by peer review) is the author/funder, who has granted bioRxiv a license to display the preprint in perpetuity. It is made available under aCC-BY 4.0 International license.

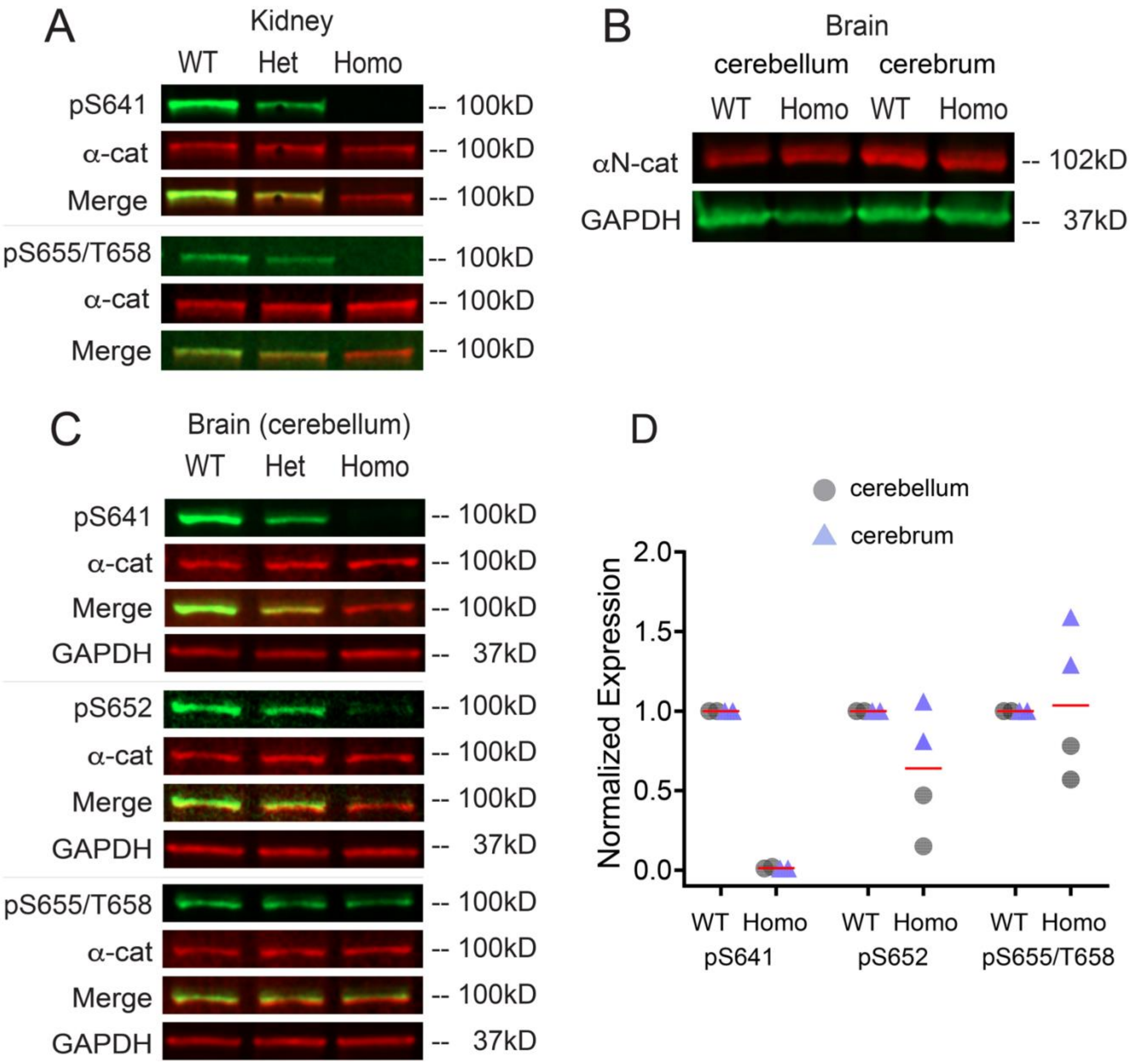


799
